# Axin Regulates Dendritic Spine Morphogenesis through Cdc42-Dependent Signaling

**DOI:** 10.1371/journal.pone.0133115

**Published:** 2015-07-23

**Authors:** Yu Chen, Zhuoyi Liang, Erkang Fei, Yuewen Chen, Xiaopu Zhou, Weiqun Fang, Wing-Yu Fu, Amy K. Y. Fu, Nancy Y. Ip

**Affiliations:** 1 Division of Life Science, State Key Laboratory of Molecular Neuroscience and Molecular Neuroscience Center, The Hong Kong University of Science and Technology, Clear Water Bay, Kowloon, Hong Kong, China; 2 Guangdong Key Laboratory of Brain Science, Disease and Drug Development, HKUST Shenzhen Research Institute, Shenzhen, Guangdong, China; University of South Alabama, UNITED STATES

## Abstract

During development, scaffold proteins serve as important platforms for orchestrating signaling complexes to transduce extracellular stimuli into intracellular responses that regulate dendritic spine morphology and function. Axin (“axis inhibitor”) is a key scaffold protein in canonical Wnt signaling that interacts with specific synaptic proteins. However, the cellular functions of these protein–protein interactions in dendritic spine morphology and synaptic regulation are unclear. Here, we report that Axin protein is enriched in synaptic fractions, colocalizes with the postsynaptic marker PSD-95 in cultured hippocampal neurons, and interacts with a signaling protein Ca^2+^/calmodulin-dependent protein kinase II (CaMKII) in synaptosomal fractions. Axin depletion by shRNA in cultured neurons or intact hippocampal CA1 regions significantly reduced dendritic spine density. Intriguingly, the defective dendritic spine morphogenesis in Axin-knockdown neurons could be restored by overexpression of the small Rho-GTPase Cdc42, whose activity is regulated by CaMKII. Moreover, pharmacological stabilization of Axin resulted in increased dendritic spine number and spontaneous neurotransmission, while Axin stabilization in hippocampal neurons reduced the elimination of dendritic spines. Taken together, our findings suggest that Axin promotes dendritic spine stabilization through Cdc42-dependent cytoskeletal reorganization.

## Introduction

Cognitive functions are believed to be encoded by a plethora of biological processes within neurons, such as the structural changes of dendritic spines harboring the postsynaptic apparatus of excitatory synapse, enrichment of synaptic components, and electrochemical transmission across synapses. The tight control and proper coordination of the signaling events underlying these processes are critical for learning and memory. Aberrant activation or inhibition of synaptic signaling is associated with various neurological disorders [1].

Synaptic scaffold proteins play a pivotal role in the spatiotemporal orchestration of signaling molecules [2]. One key postsynaptic scaffold is postsynaptic density-95 (PSD-95), which provides docking sites for cell surface ion channels and neurotransmitter receptors, transducing extracellular stimuli into intracellular signaling events to control synapse morphology and function [3]. PSD-95 associates with synaptic AMPA receptors via interaction with stargazin, a transmembrane AMPA receptor regulatory protein [4]. Acute inactivation of PSD-95 reduces the surface expression of AMPA receptors, suggesting that scaffold proteins play a key role in stabilizing synaptic components [5]. Meanwhile, PSD-95 interacts with regulators of small Rho-GTPases, the guanine nucleotide exchange factor (GEF) kalirin, and the GTPase-activating protein (GAP) SNX26; this balances the polymerization and depolymerization of the actin cytoskeletal network, which underlies the development and plasticity of dendritic spines [6, 7]. However, the scaffolds responsible for coordinating the synaptic signaling events and the underlying molecular basis remain incompletely understood.

Axin (“axis inhibitor”), a scaffold protein that is well characterized in canonical Wnt signaling, regulates glycogen synthase kinase-3β (GSK-3β)–mediated β-catenin phosphorylation and degradation through interactions with different signaling components [8]. The functional involvement of Axin in the development and functioning of the nervous system is only beginning to be unraveled. For example, during embryonic neurogenesis, the cytoplasmic or nuclear localization of Axin is a key determinant of the amplification or differentiation status of intermediate progenitors, which is controlled through the phosphorylation of Axin at Thr485 by cyclin-dependent kinase 5 (Cdk5), a proline-directed serine/threonine kinase, [9]. Stabilizing Axin with the tankyrase inhibitor XAV939 *in vivo* leads to overproduction of upper-layer neurons and an imbalance between excitatory and inhibitory neurotransmission [10, 11]. In addition, the phosphorylation of Axin by Cdk5 facilitates axon formation in the developing cortex through the enhancement of Axin–GSK-3β interaction [12]. While the functions of Axin in mature neurons, specifically at synapses, are unknown, Axin has emerged as an interacting partner of several synaptic-enriched proteins such as GSK-3β, β-catenin, Adenomatous polyposis coli (APC), Dishevelled (Dvl), Grb4, and S-SCAM [13]. These observations suggest that Axin may serve as a scaffold platform that regulates synaptic functions through interactions with different proteins.

The present study revealed that Axin localizes at neuronal synapses. Loss of Axin in cultured neurons or CA1 pyramidal neurons significantly reduced dendritic spine density. Pharmacological stabilization of Axin in neurons increased the number of dendritic spines and neurotransmission. Moreover, expression of the small Rho-GTPase Cdc42 restored the dendritic spine morphology in Axin-depleted neurons. In addition, we showed that Axin interacts with Ca^2+^/calmodulin-dependent protein kinase II (CaMKII), the key protein that controls Cdc42 activity in dendritic spines. Thus, the present study reveals a novel mechanism by which Axin regulates dendritic spine morphogenesis via Cdc42-mediated cytoskeletal reorganization.

## Materials and Methods

### Animals

Rats and mice were bred in the Animal and Plant Care Facility of The Hong Kong University of Science and Technology and handled in accordance with the Animals (Control of Experiments) Ordinance of Hong Kong. All animal experiments were performed in accordance with protocols #2009056 and #2009012 approved by the Animal Care Committee of the Hong Kong University of Science and Technology.

### Constructs and antibodies

The construct expressing Axin shRNA (target sequence: 5′-AGUACAUCCUGGAUAGCAA-3′) was prepared as described previously [12]. The RNAi-resistant form of Axin was generated as described previously and subcloned into the pcDNA3 vector [9]. Antibodies against PSD-95, GluA1, and pSer831-GluA1 were purchased from Cell Signaling Technology; β-actin was purchased from Sigma; and GFP IgG2a was purchased from Invitrogen Life Technologies. Custom antibodies for detecting pThr485 Axin and total Axin were generated and prepared as described previously [12]. Alexa Fluor 488 and 546 conjugated secondary antibodies were purchased from Life Technologies, and HRP-conjugated anti-mouse and anti-rabbit secondary antibodies were purchased from Cell Signaling Technology.

### Preparation of postsynaptic density, cytosolic, and nuclear fractions

The postsynaptic density (PSD) fraction was prepared as described previously [14]. Briefly, mouse brains were homogenized in HEPES buffer (0.32 M sucrose, 4 mM HEPES [pH 7.4]). The homogenate (Hom.) was centrifuged to remove the pelleted nuclear fraction (P1), and the supernatant was centrifuged again to yield crude synaptosomal fraction (P2). The washed P2 fraction (P2′) was subjected to hypoosmotic shock and lysis before centrifugation again. The resultant pellet was resuspended and centrifuged in a sucrose gradient to yield the synaptic plasma member (SPM) fraction. The PSD fraction was extracted from the SPM fraction with 0.5% Triton X-100. Cytosolic and nuclear fractions were prepared using the Nuclear Complex Co-IP Kit (Active Motif).

### Mass spectrometry analysis

Whole mouse brains were homogenized, and the washed synaptosomal fraction (P2′) was prepared as described above. The pellet was resuspended, and 2 mg protein was subjected to the co-immunoprecipitation assay with Axin antibody. Normal rabbit IgG was used as a control. The co-immunoprecipitates were resolved by SDS-PAGE. Trypsin digestion and peptide recovery from the gels were performed, followed by mass spectrometry analysis by Shanghai Applied Protein Technology (Shanghai, China). Proteins with a unique peptide count ≥2 were considered positive. Only proteins with a unique peptide count >5 are shown in Table 1.

**Table 1:** Candidates of Axin-interacting proteins in mouse brain synaptosomal fraction.

	Protein Identity	Database/Accession number	Unique Peptide Count^a^	Protein Coverage%	MW/pI (kDa)
**1**	Clathrin heavy chain 1	Q68FD5	23	13.79	191.6/5.5
**2**	Stress-70 protein, mitochondrial	P38647	22	37.41	73.5/5.8
**3**	Spna2 protein	B2RXX6	18	7.31	285.2/5.2
**4**	Spectrin alpha chain, non-erythrocytic 1	E9Q447	18	7.30	285.3/5.2
**5**	Heat shock-related 70 kDa protein 2	P17156	17	25.59	69.6/5.5
**6**	Heat shock protein 90, alpha (Cytosolic), class A member 1	Q80Y52	16	19.37	84.8/4.9
**7**	Spectrin beta chain, non-erythrocytic 1	Q62261	14	5.37	274.2/5.4
**8**	Heat shock protein 84b	Q71LX8	13	16.71	83.3/5.0
**9**	Desmoplakin	E9Q557	13	4.06	332.9/6.4
**10**	Neurofilament light polypeptide	P08551	13	22.10	61.5/4.6
**11**	Neurofilament medium polypeptide	P08553	12	13.80	95.9/4.8
**12**	Sodium/potassium-transporting ATPase subunit alpha-3	Q6PIC6	9	10.17	111.7/5.3
**13**	Sodium/potassium-transporting ATPase subunit alpha-2	Q6PIE5	9	10.10	112.2/5.4
**14**	Heat shock protein 1-like	A1L347	8	13.26	70.6/5.9
**15**	Calcium/calmodulin-dependent protein kinase type II subunit beta	Q5SVI3	8	16.22	58.1/6.6
**16**	Alpha-internexin	P46660	8	15.77	55.4/5.4
**17**	Sodium/potassium-transporting ATPase subunit alpha-1	Q8VDN2	8	8.99	113.0/5.3
**18**	Calcium/calmodulin-dependent protein kinase type II subunit alpha	F8WIS9	7	14.11	55.3/7.1
**19**	Transforming acidic coiled-coil-containing protein 1	Q6Y685	6	7.75	84.0/5.0
**20**	Heat shock protein 105 kDa	E9Q0U7	6	8.32	91.7/5.5
**21**	Tyrosine-protein phosphatase non-receptor type 11	P35235	6	9.05	68.5/6.9
**22**	Dynamin-1	P39053	6	6.34	97.8/7.6
**23**	Guanine nucleotide-binding protein subunit beta-2-like 1	P68040	6	22.08	35.1/7.6
**24**	Microtubule-associated protein 6	Q7TSJ2	6	7.84	96.4/9.5

^a^Only proteins with the unique peptide count >5 are shown.

### Cell culture and transfection

Cultures of primary hippocampal and cortical neurons were prepared from embryonic day (E) 18 Sprague–Dawley rats as described previously [15] and maintained in Neurobasal medium (Life Technologies) plus 2% B27 supplement (Life Technologies). DMSO-dissolved XAV939 was diluted in the culture medium before being applied to the neuronal cultures. Constructs encoding Axin shRNA, wild-type Axin, and its mutants were delivered into primary hippocampal neurons by calcium phosphate transfection [15].

### Electrophysiological recording

Hippocampal neurons were treated with 5 μm XAV939 at 17 days *in vitro* (DIV) for 2 h or 3 days. Spontaneous miniature excitatory postsynaptic currents (mEPSCs) were measured at a holding voltage of −70 mV. Picrotoxin (200 μm) and tetrodotoxin (0.5 μm) were used to block inhibitory synaptic transmission and EPSCs evoked by action potentials, respectively. The experiments were repeated 3 times with at least 10 neurons for each condition each time. Data are presented as mean ± SEM. Statistical analysis was performed using Student’s *t*-test.

### Virus injection and tissue processing

Lentiviral particles expressing Axin shRNA (pFUGW-shAxin) were packaged at the Gene Transfer Vector Core of the University of Iowa (Iowa City, IA, USA) and delivered into the hippocampal CA1 region of 2-month-old mice by using the stereotactic apparatus and Quintessential Stereotaxic Injector (Stoelting) as described previously [16]. Mice were anesthetized with 2% isoflurane before the surgery, and opthalmic ointment was used to prevent eye drying. Antibiotics were injected subcutaneously upon completion of surgery, and the mice recovered under a heat lamp.

The mice were sacrificed 3 weeks after virus injection and subjected to cardiac perfusion with 4% paraformaldehyde after anesthetized by ketamine. The forebrains were dissected, post-fixed overnight in 4% paraformaldehyde, and cut into 80-μm-thick coronal sections by a Leica VT1000 S vibrating blade microtome.

### Immunostaining, confocal imaging, and quantification

Primary hippocampal neurons were fixed with 4% paraformaldehyde plus 4% sucrose for 20 min and incubated with primary antibodies overnight at 4°C, followed by secondary antibodies at room temperature for 1 h. Images were taken under a Nikon A1 scanning confocal microscope with a 40× or 60× oil immersion objective. Mouse brain sections were stained with anti-GFP antibody in Tris-buffered saline containing 0.1% Triton X-100 and 3% bovine serum albumin. Images of the hippocampal region were captured using a Leica TCS SP8 confocal microscope with a 40× oil immersion objective. The number, length, and complexity of neuronal dendrites were quantified using ImageJ (National Institute of Health, Bethesda, MA, USA) with the NeuronJ [17] and Sholl analysis plugins (Anirvan Ghosh). Dendritic spines were quantified using Metamorph 7.0 (Molecular Devices). All data are presented as mean ± SEM. Statistical comparisons were performed using Student’s *t*-test and one-way ANOVA.

## Results

### Axin localizes at neuronal synapses and interacts with CaMKII

**Fig 1:**
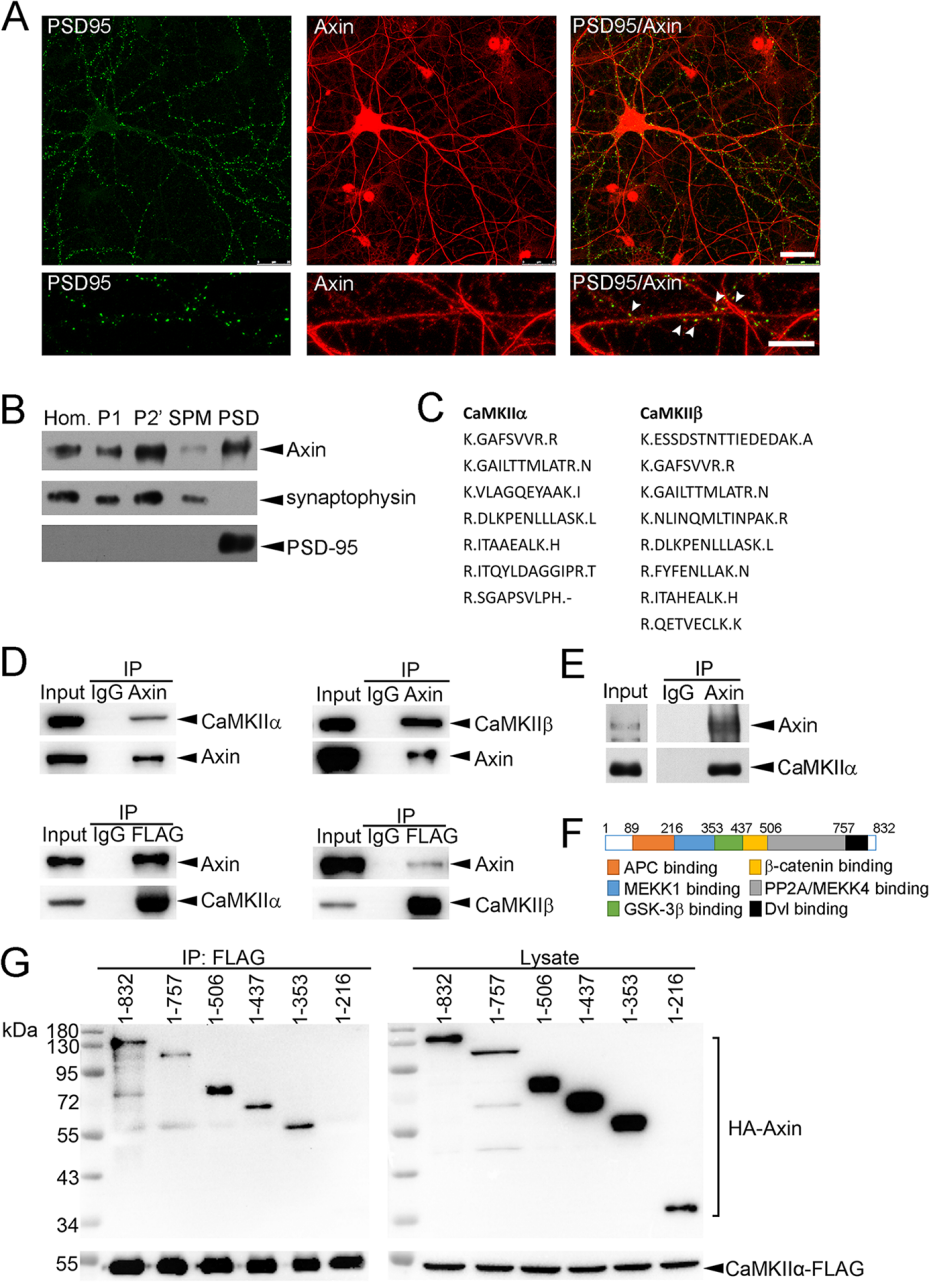
Axin is expressed at neuronal synapses. (A) Axin co-localized with PSD-95 puncta in hippocampal neurons. Hippocampal neurons (20 DIV) were stained with antibodies against Axin and PSD-95. Upper panels: representative images. Scale bar: 25 μm. Lower panels: higher-magnification images showing Axin colocalization with PSD-95 at synapses. Scale bar: 10 μm. (B) Axin was readily detected in the P2′, SPM, and PSD fractions prepared from mouse brains. PSD-95 and synaptophysin are pre- and postsynaptic markers, respectively. Hom: homogenate; P1: nuclear fractions; P2′: crude synaptosomal fraction; SPM: synaptic plasma membrane; PSD: postsynaptic density. (C) Mass spectrometry analysis identified unique peptides representing CaMKIIα and CaMKIIβ in the mouse brain synaptosomal fraction pulled down by Axin antibody. (D) Co-immunoprecipitation assay demonstrated that Axin strongly associated with CaMKIIα and CaMKIIβ in HEK293T cells. (E) CaMKIIα was co-immunoprecipitated with Axin from the mouse brain synaptosomal fraction. (F) Schematic structure of Axin protein. (G) Amino acids 216–353 of Axin were important for Axin and CaMKIIα interaction.

To investigate the roles of Axin in synapse development and/or functioning, we first examined its expression profile in primary rat hippocampal neurons at 20 DIV. Axin was strongly expressed in cell soma and dendrites, and Axin puncta along the dendrites were co-localized with PSD-95 (Fig 1A). In 1-month-old mouse brains, Axin was detected in the P2′ and SPM fractions, which contain both presynaptic and postsynaptic components (Fig 1B). Axin was strongly expressed in the PSD fraction, indicating the enrichment of Axin protein in the postsynaptic compartment (Fig 1B). Mass spectrometry screening for Axin-interacting proteins in the mouse brain synaptosomal fraction produced a list of candidates, some of which have been shown to regulate synaptic functions (Table 1). Seven and eight unique peptides for CaMKIIα and CaMKIIβ were identified in the co-immunoprecipitates, respectively (Fig 1C). Co-immunoprecipitation assay in HEK293T cells revealed strong interactions between Axin and CaMKIIα/CaMKIIβ (Fig 1D). The *in vivo* interaction between Axin and CaMKIIα was observed in the mouse brain synaptosomal fraction (Fig 1E) as well as the P1 (i.e., pelleted nuclear fraction) and S2 (i.e., cytosol and light membranes) fractions (data not shown). We further identified that amino acids 216–353 of Axin, a region overlapping with MEKK1-binding domain, were important for Axin-CaMKIIα interaction (Fig 1F and 1G). These findings suggest that Axin localizes at the postsynaptic apparatus and therefore might play an important role as a signaling scaffold to shape synaptic structures and functions.

### Axin regulates dendritic spine morphogenesis via Cdc42

**Fig 2:**
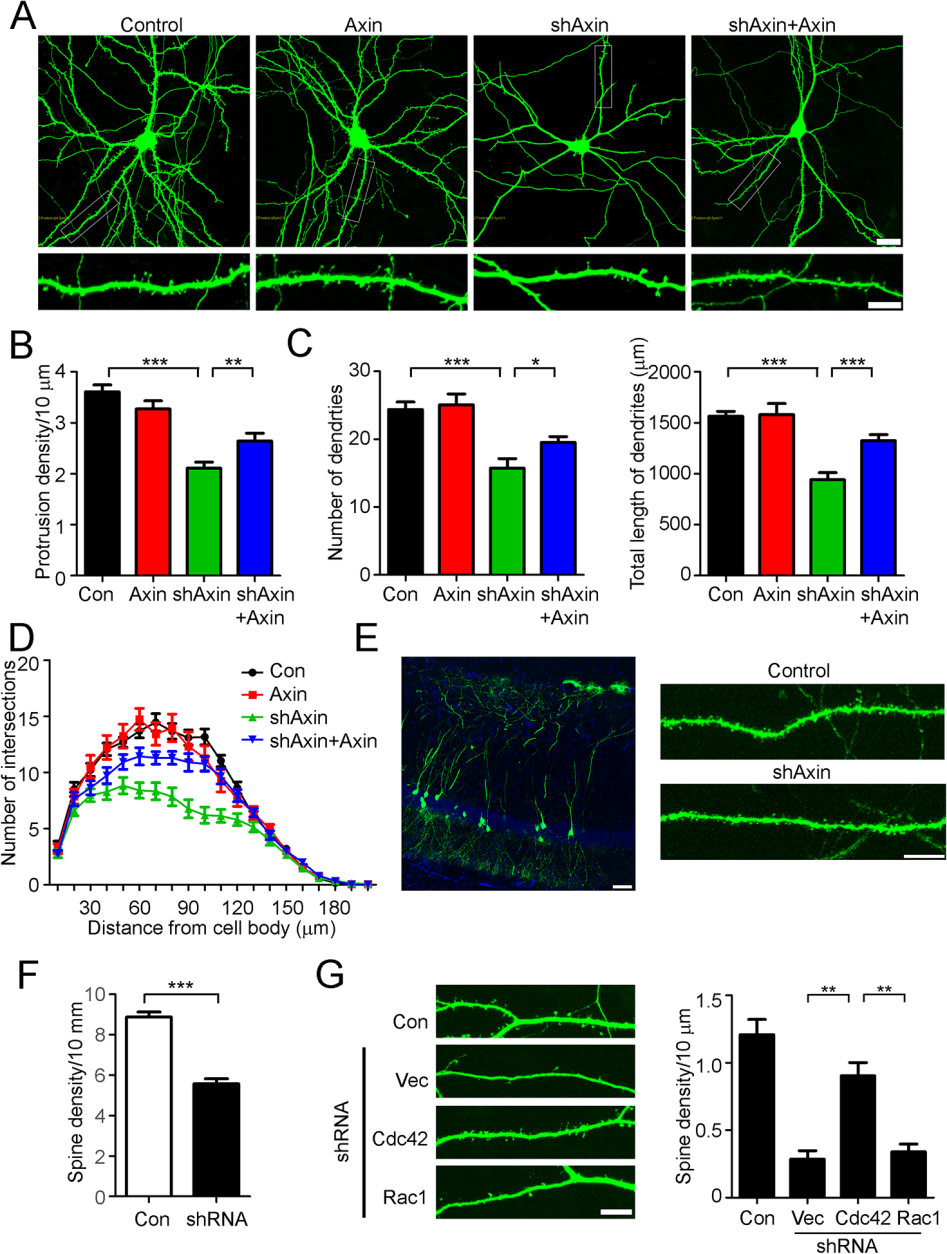
Axin is required for dendritic spine morphogenesis. (A–D) Axin knockdown led to the simplification of dendritic trees and reduction of dendritic spine number. (A) Upper panels: representative images showing hippocampal neuron morphology. Scale bar: 20 μm. Lower panels: higher-magnification images showing dendritic spines morphology. Scale bar: 10 μm. (B) Axin knockdown significantly reduced protrusion density, which was partially rescued by re-expressing the RNAi-resistant form of Axin. One-way ANOVA, *n* = 45, **p < 0.01 shAxin vs shAxin+Axin, ***p < 0.001 shAxin vs Con. (C) The number and total length of dendrites in Axin-knockdown neurons were reduced. One-way ANOVA, *n* = 15, *p < 0.05 shAxin vs shAxin+Axin, ***p < 0.001 shAxin vs Con. (D) Sholl analysis showed that the complexity of dendritic trees was reduced in Axin-knockdown neurons. *n* = 15. (E) Lentiviral knockdown of Axin in the hippocampal CA1 region reduced dendritic spine density. Left panel: representative image showing virus-infected neurons in the hippocampal CA1 region. Scale bar: 50 μm. Right panels: higher-magnification images showing the dendritic spines along dendrites. Scale bar: 10 μm. (F) Silencing Axin significantly reduced dendritic spine density in the CA1 region. Student’s *t*-test; GFP, *n* = 56; shRNA, *n* = 24; ***p < 0.001. (G) Overexpression of Cdc42 but not Rac1 rescued the defective dendritic spine phenotype in Axin-knockdown neurons. Left panels: representative images showing the dendritic morphology. Right panels: quantitation of dendritic spine density. Scale bar: 10 μm; one-way ANOVA, *n* = 15, **p < 0.01 shRNA+vector vs shRNA+Cdc42; shRNA+Rac1 vs shRNA+Cdc42.

To specifically examine the role of Axin dendritic spine morphogenesis, Axin protein was knocked down by shRNA in cultured hippocampal neurons at 17 DIV. Silencing Axin expression significantly reduced dendritic spine density (by ~36%; Fig 2A and 2B). The reduction of dendritic spine density in Axin-knockdown neurons was partially rescued by the re-expression of RNAi-resistant wild-type Axin, suggesting that Axin has a critical role in dendritic spine maintenance (Fig 2A and 2B). Furthermore, Axin depletion also reduced the complexity of dendritic trees, as demonstrated by decreased dendrite number and total dendrite length in Axin-knockdown neurons (Fig 2C and 2D). We further confirmed the essential role of Axin in shaping dendritic spines in the mouse hippocampus. Axin knockdown in the CA1 region *in vivo* significantly reduced dendritic spine density (by ~37%; Fig 2E and 2F).

Actin cytoskeletal dynamics are crucial for the structural changes of dendritic spines, which are regulated by various signaling molecules including small Rho GTPases RhoA, Cdc42, and Rac1 [18]. In particular, Cdc42 and Rac1 are implicated in stabilizing actin filaments in dendritic spines, while RhoA activation leads to spine retraction by destabilizing the actin network. Accordingly, the expression of Cdc42 but not Rac1 in Axin-knockdown neurons restored the dendritic spine density, indicating that Cdc42 is involved in Axin-dependent dendritic spine morphogenesis (Fig 2G). As Cdc42 activation within dendritic spines depends on CaMKII [19], these findings suggest that Axin plays an important role in scaffolding CaMKII in dendritic spines for Cdc42-mediated cytoskeletal reorganization.

### Stabilization of endogenous Axin increases dendritic spine stability

**Fig 3:**
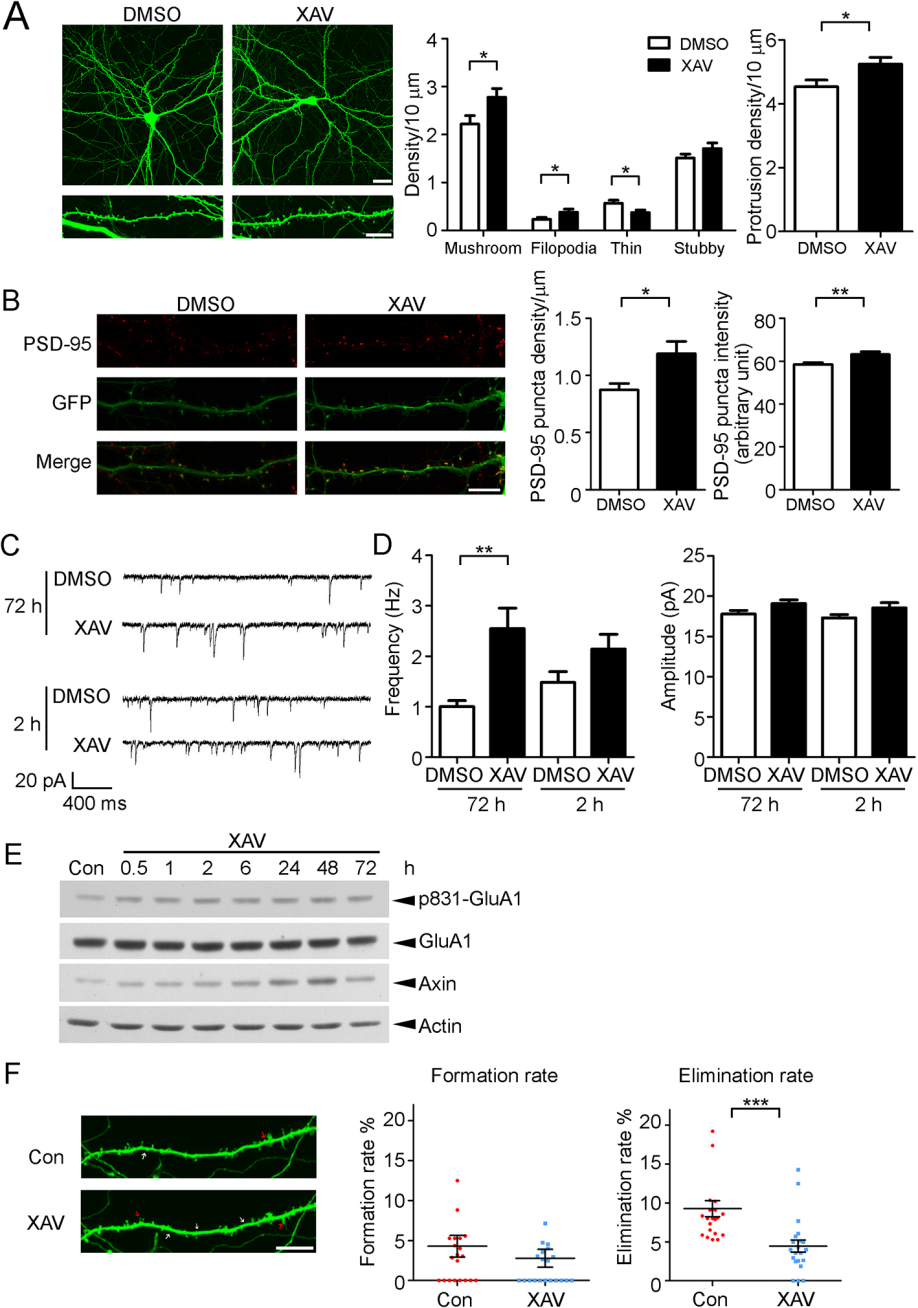
Axin stabilization increases dendritic spine density and synaptic transmission. (A) Treating hippocampal neurons (17 DIV) with the Axin stabilizer XAV939 for 3 days significantly increased the density of mature dendritic spines and total protrusions. Scale bar: upper panels = 20 μm, lower panels = 10 μm; Student’s *t*-test, *n* = 24, *p < 0.05. (B) XAV939 treatment increased PSD-95–positive puncta along dendrites. Left panels: representative images showing the dendritic morphology of control and XAV939-treated neurons. Right panels: quantitation of PSD-95 puncta density and intensity. Scale bar: 10 μm; Student’s *t*-test, *n* = 15, *p < 0.05, **p < 0.01. (C) Representative mEPSC traces of control and XAV939-treated neurons. (D) XAV939 treatment increased the frequency but not the amplitude of mEPSCs in hippocampal neurons. One-way ANOVA, *n* = 22, **p < 0.01 vs DMSO for 72 h. (E) Cortical neurons were treated with XAV939 for the indicated times. Enhanced GluA1 phosphorylation at Ser831 was observed from 0.5–72 h after treatment. (F) Live imaging demonstrated that XAV939 treatment did not induce an obvious change in the formation rate of dendritic spines but significantly reduced their elimination rate. Left panels: representative images showing spine morphology in cultured neurons. Right panels: quantitative results of spine formation/elimination rate. Scale bar: 10 μm; Student’s *t*-test, *n* = 21, ***p < 0.001.

The tankyrase inhibitor XAV939 can pharmacologically stabilize endogenous Axin in various cell lines and developing mammalian brains [11]. We confirmed the efficacy of XAV939 to stabilize Axin in cultured neurons [9]; accordingly, treating neurons with XAV939 at 17 DIV for 3 days significantly increased the density of mushroom-shaped mature dendritic spines as well as the total number of protrusions along the dendrites (Fig 3A). Furthermore, there were significantly more postsynaptic marker PSD-95–positive clusters along the dendrites in XAV939-treated neurons, suggesting that XAV939 increases the number of synapses (Fig 3B). Consistently, hippocampal neurons treated with XAV939 for 2 h tended to exhibit a higher spontaneous mEPSC frequency, which can be attributed at least in part to the increased numbers of dendritic spines and synapses in these neurons. Although presynaptic axons are enriched with Axin during early development [12], further investigation is needed to determine if XAV939 increases the probability of presynaptic neurotransmitter release to activate postsynaptic glutamate receptors. The XAV939-induced increase of mEPSC frequency became significant at 72 h, whereas the amplitude of mEPSCs, which reflects the abundance of AMPA receptors, remained unchanged (Fig 3C and 3D). These findings suggest that elevated Axin levels increase the number of functional excitatory synapses in hippocampal neurons, consequently enhancing neurotransmission. Interestingly, XAV939 induced the CaMKII-dependent phosphorylation of GluA1 (an AMPA receptor subunit) at Ser831 in a dose-dependent manner. The effect of XAV939 on GluA1 Ser831 phosphorylation was observed as early as 0.5 h after treatment and persisted for at least 3 days (Fig 3E), while the functional role of XAV939-induced GluA1 phosphorylation requires further characterization. Furthermore, we used a live-imaging approach to examine how Axin stabilization regulates dendritic spine morphology. Interestingly, XAV939 treatment decreased spine elimination, whereas spine formation remained basically unchanged. This resulted in a net increase of dendritic spines, corroborating the idea that Axin is required for dendritic spine stability (Fig 3F).

## Discussion

Dendritic spine morphology and the related underlying synaptic functions are controlled temporally and spatially by well-organized molecular complexes. The present study demonstrates that Axin, a key scaffolding protein, is essential for dendritic spine morphogenesis by orchestrating the intracellular signaling complex, leading to cytoskeletal reorganization.

Despite a lack of direct experimental evidence, Axin has long been suggested to affect synapses [8, 13]. A recent high-throughput screening of a lentiviral RNAi library revealed that Axin preferentially regulates the synaptogenesis of excitatory synapses from 4–14 DIV; moreover, Axin depletion leads to a reduction of PSD-95 puncta [20]. In the present study, in more mature neurons at 20 DIV, transient stabilization of Axin in neurons increased the number of PSD-95 clusters and reduced the elimination rate of dendritic spines (Fig 3B and 3F). These findings indicate that Axin is required not only for synapse formation, but also for synapse maintenance.

More importantly, the present study revealed the signaling pathways by which Axin exerts its function at synapses. CaMKII, a multifaceted synaptic Axin-interacting protein, regulates the synaptic structural and functional plasticity through a complex signaling network involving molecules such as small Rho-GTPases and cell surface neurotransmitter receptors [21]. Our results suggest that Axin preferentially acts through the small Rho-GTPase Cdc42 in dendritic spine morphogenesis, which is concordant with the report that glutamate uncaging-induced Cdc42 activation and spine growth are diminished by the CaMKII inhibitors KN62 and AIP2 [19]. Axin may function as a scaffold to anchor CaMKII and restrict the local activation of Cdc42 within the dendritic spines [19]. In addition to the structural plasticity of dendritic spines, CaMKII regulates the phosphorylation of the neurotransmitter receptor subunit GluA1 at Ser831, an important modification for receptor trafficking to the synapse and its conductance [22]. Stabilizing endogenous Axin augments Ser831 phosphorylation, supporting the idea that Axin provides a docking site for CaMKII to potentiate synaptic functions. Understanding the interaction domains of Axin and CaMKII as well as their regulatory mechanisms will be important for understanding the scaffolding role of Axin in the structural and functional changes of synapses. On the other hand, Axin–S-SCAM interaction provides an alternative pathway by which the AMPA receptor can dock at synapses via stargazing [23, 24], providing a possible molecular basis that underlies the action of Axin in stabilizing synaptic neurotransmitter receptors.

Small Rho-GTPases such as Cdc42, Rac1, and RhoA are believed to play important roles in morphological and functional changes of dendritic spines by modulating the balance between actin monomers and filaments [25]. As a downstream effector of Axin, the activity of Cdc42 is tightly controlled by GEFs such as intersectin1 and β-PIX, whose regulations of dendritic spine morphology are well characterized [26–28]. However, neither the GEF nor the counteracting GAP that directly associates with Axin has been identified. Rac/Cdc42-specific GEF β-PIX is a candidate component in the Axin complex through anchorage via β-catenin and cadherin [27]. Nonetheless, further research is required to characterize the mechanisms by which Axin scaffolds GEFs to promote Cdc42 activity within dendritic spines.

Other synaptic proteins that anchor the Axin-based scaffold include growth factor receptor-bound protein 4 (Grb4), S-SCAM, and some components in canonical Wnt signaling [13]. Postsynaptic Grb4 associates with G-protein-coupled receptor kinase-interacting protein 1 (GIT1) upon activation of ephrin-B, a ligand of Eph receptors that can transduce bidirectional synaptic signaling [29, 30]. By mediating the reverse signaling as a receptor, ephrin-B shapes the structural maturation and functional plasticity of neuronal synapses; however, the downstream mechanisms are not fully understood [31]. As GIT associates with the GEF β-PIX, it is interesting to speculate that Axin/Grb4 complex plays a role in recruiting β-PIX/GIT upon ephrin-B activation, thus transducing ephrin-B signaling into small Rho-GTPase activation in dendritic spines [27, 32]. Although the domains through which Axin interacts with most of its partners are known, investigation using super-resolution imaging is required to elucidate the spatiotemporal orchestration of the Axin complex at synapses.

Changes of dendritic spine morphology and function are implicated in the regulation of synaptic strength. A short period of high-frequency stimulation can induce long-term enhancement of synaptic strength; this is termed long-term potentiation (LTP) and is believed to be associated with memory processes. CaMKII activation by activity-dependent calcium influx and its subsequent translocation to synapses are key events in the early stage of LTP [33]. CaMKII can also be stimulated by the Wnt signaling that facilitates LTP [34], raising the intriguing question of whether Axin can serve as a scaffold to couple the Wnt components to CaMKII activation during LTP. Thus, manipulating Axin expression in specific neuronal circuits (e.g., the hippocampal CA3–CA1 pathway) through conditional knockout or virus-mediated knockdown would help elucidate its role in the regulation of synaptic plasticity.
